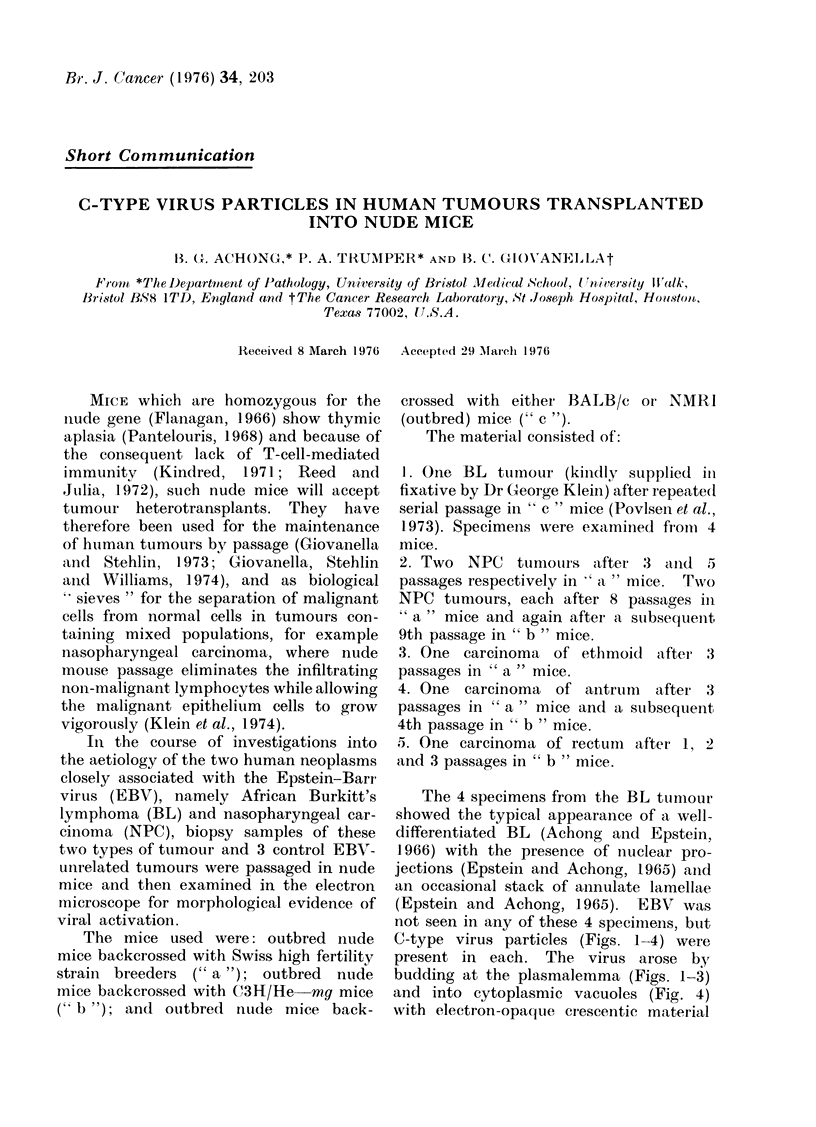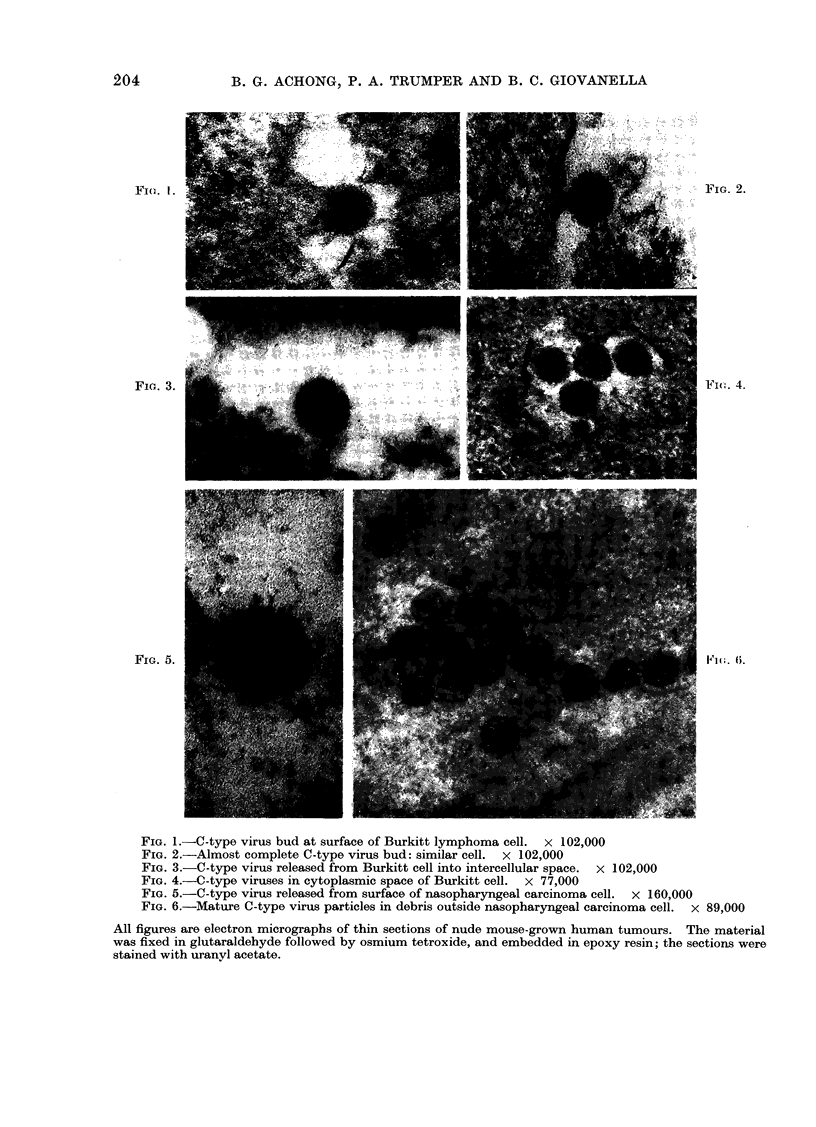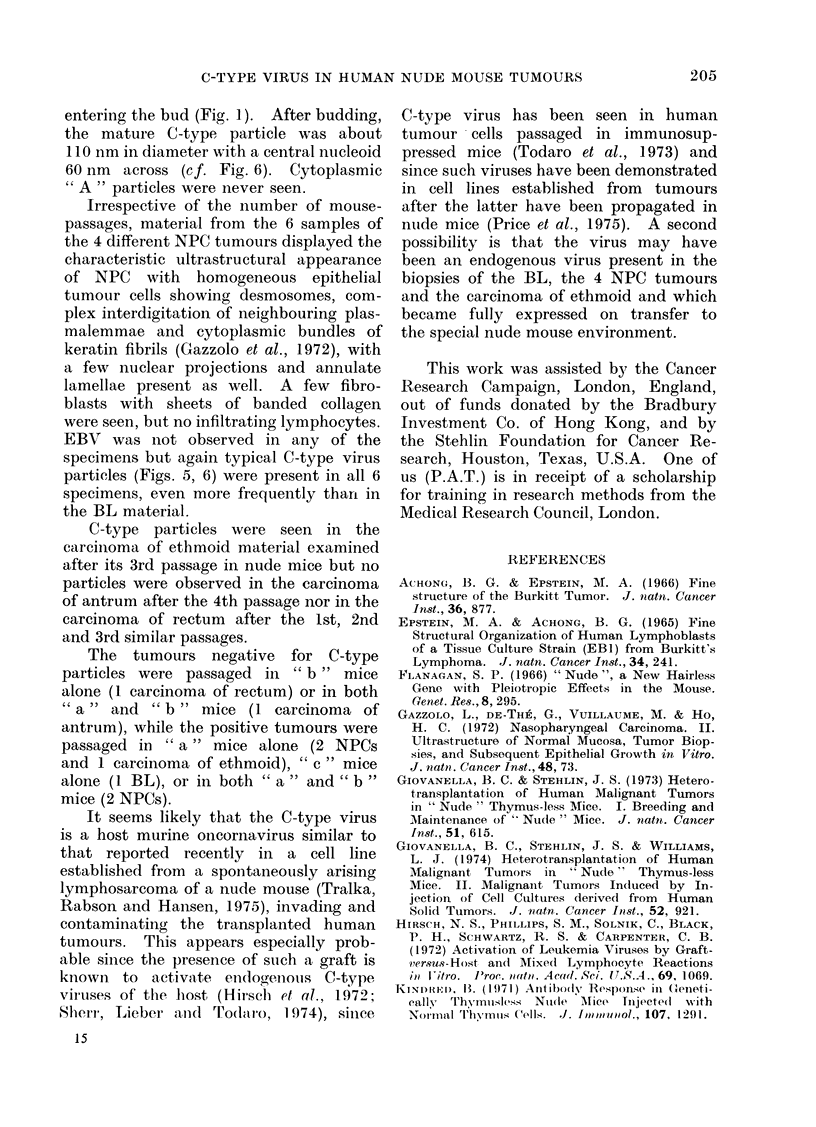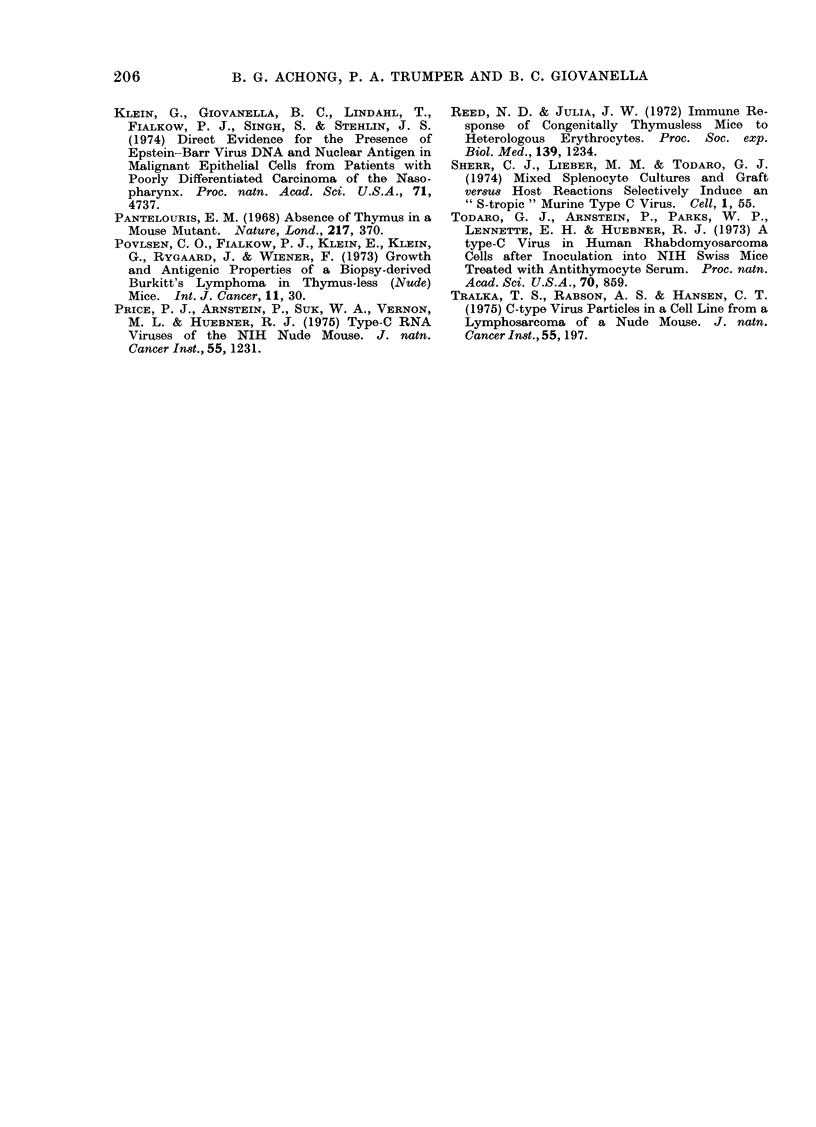# C-type virus particles in human tumours transplanted into nude mice.

**DOI:** 10.1038/bjc.1976.144

**Published:** 1976-08

**Authors:** B. G. Achong, P. A. Trumper, B. C. Giovanella

## Abstract

**Images:**


Br. J. Cancer (1976) **34**, 203

***Short Communication***

### C-TYPE VIRUS PARTICLES IN HUMAN TUMOURS TRANSPLANTED INTO NUDE MICE

B. G. ACHONG,* P. A. TRUMPER* AND B. C. GIOVANELLA†

*From *The Department of Pathology, University of Bristol Medical School, University Walk, Bristol BS8 1TD, England and †The Cancer Research Laboratory, St Joseph Hospital, Houston, Texas* 77002, *U.S.A.*

Received 8 March 1976 Accepted 29 March 1976

MICE which are homozygous for the nude gene (Flanagan, 1966) show thymic aplasia (Pantelouris, 1968) and because of the consequent lack of T-cell-mediated immunity (Kindred, 1971; Reed and Julia, 1972), such nude mice will accept tumour heterotransplants. They have therefore been used for the maintenance of human tumours by passage (Giovanella and Stehlin, 1973; Giovanella, Stehlin and Williams, 1974), and as biological " sieves " for the separation of malignant cells from normal cells in tumours containing mixed populations, for example nasopharyngeal carcinoma, where nude mouse passage eliminates the infiltrating non-malignant lymphocytes while allowing the malignant epithelium cells to grow vigorously (Klein *et al.*, 1974).

In the course of investigations into the aetiology of the two human neoplasms closely associated with the Epstein–Barr virus (EBV), namely African Burkitt's lymphoma (BL) and nasopharyngeal carcinoma (NPC), biopsy samples of these two types of tumour and 3 control EBV-unrelated tumours were passaged in nude mice and then examined in the electron microscope for morphological evidence of viral activation.

The mice used were: outbred nude mice backcrossed with Swiss high fertility strain breeders (" a "); outbred nude mice backcrossed with C3H/He—*mg* mice (" b "); and outbred nude mice backcrossed with either BALB/c or NMRI (outbred) mice (" c ").

The material consisted of:

1. One BL tumour (kindly supplied in fixative by Dr George Klein) after repeated serial passage in " c " mice (Povlsen *et al.*, 1973). Specimens were examined from 4 mice.
2. Two NPC tumours after 3 and 5 passages respectively in " a " mice. Two NPC tumours, each after 8 passages in " a " mice and again after a subsequent 9th passage in " b " mice.
3. One carcinoma of ethmoid after 3 passages in " a " mice.
4. One carcinoma of antrum after 3 passages in " a " mice and a subsequent 4th passage in " b " mice.
5. One carcinoma of rectum after 1, 2 and 3 passages in " b " mice.

The 4 specimens from the BL tumour showed the typical appearance of a well-differentiated BL (Achong and Epstein, 1966) with the presence of nuclear projections (Epstein and Achong, 1965) and an occasional stack of annulate lamellae (Epstein and Achong, 1965). EBV was not seen in any of these 4 specimens, but C-type virus particles (Figs. 1–4) were present in each. The virus arose by budding at the plasmalemma (Figs. 1–3) and into cytoplasmic vacuoles (Fig. 4) with electron-opaque crescentic material

FIG. 1.

FIG. 2.

FIG. 3.

FIG. 4.

FIG. 5.

FIG. 6.

FIG. 1.—C-type virus bud at surface of Burkitt lymphoma cell. × 102,000
FIG. 2.—Almost complete C-type virus bud: similar cell. × 102,000
FIG. 3.—C-type virus released from Burkitt cell into intercellular space. × 102,000
FIG. 4.—C-type viruses in cytoplasmic space of Burkitt cell. × 77,000
FIG. 5.—C-type virus released from surface of nasopharyngeal carcinoma cell. × 160,000
FIG. 6.—Mature C-type virus particles in debris outside nasopharyngeal carcinoma cell. × 89,000

All figures are electron micrographs of thin sections of nude mouse-grown human tumours. The material was fixed in glutaraldehyde followed by osmium tetroxide, and embedded in epoxy resin; the sections were stained with uranyl acetate.

entering the bud (Fig. 1). After budding, the mature C-type particle was about 110 nm in diameter with a central nucleoid 60 nm across (*cf.* Fig. 6). Cytoplasmic " A " particles were never seen.

Irrespective of the number of mouse-passages, material from the 6 samples of the 4 different NPC tumours displayed the characteristic ultrastructural appearance of NPC with homogeneous epithelial tumour cells showing desmosomes, complex interdigitation of neighbouring plasmalemmae and cytoplasmic bundles of keratin fibrils (Gazzolo *et al.*, 1972), with a few nuclear projections and annulate lamellae present as well. A few fibroblasts with sheets of banded collagen were seen, but no infiltrating lymphocytes. EBV was not observed in any of the specimens but again typical C-type virus particles (Figs. 5, 6) were present in all 6 specimens, even more frequently than in the BL material.

C-type particles were seen in the carcinoma of ethmoid material examined after its 3rd passage in nude mice but no particles were observed in the carcinoma of antrum after the 4th passage nor in the carcinoma of rectum after the 1st, 2nd and 3rd similar passages.

The tumours negative for C-type particles were passaged in " b " mice alone (1 carcinoma of rectum) or in both " a " and " b " mice (1 carcinoma of antrum), while the positive tumours were passaged in " a " mice alone (2 NPCs and 1 carcinoma of ethmoid), " c " mice alone (1 BL), or in both " a " and " b " mice (2 NPCs).

It seems likely that the C-type virus is a host murine oncornavirus similar to that reported recently in a cell line established from a spontaneously arising lymphosarcoma of a nude mouse (Tralka, Rabson and Hansen, 1975), invading and contaminating the transplanted human tumours. This appears especially probable since the presence of such a graft is known to activate endogenous C-type viruses of the host (Hirsch *et al.*, 1972; Sherr, Lieber and Todaro, 1974), since C-type virus has been seen in human tumour cells passaged in immunosuppressed mice (Todaro *et al.*, 1973) and since such viruses have been demonstrated in cell lines established from tumours after the latter have been propagated in nude mice (Price *et al.*, 1975). A second possibility is that the virus may have been an endogenous virus present in the biopsies of the BL, the 4 NPC tumours and the carcinoma of ethmoid and which became fully expressed on transfer to the special nude mouse environment.

This work was assisted by the Cancer Research Campaign, London, England, out of funds donated by the Bradbury Investment Co. of Hong Kong, and by the Stehlin Foundation for Cancer Research, Houston, Texas, U.S.A. One of us (P.A.T.) is in receipt of a scholarship for training in research methods from the Medical Research Council, London.

### REFERENCES

Achong, B. G. & Epstein, M. A. (1966) Fine structure of the Burkitt Tumor. *J. natn. Cancer Inst.*, **36**, 877.

Epstein, M. A. & Achong, B. G. (1965) Fine Structural Organization of Human Lymphoblasts of a Tissue Culture Strain (EB1) from Burkitt's Lymphoma. *J. natn. Cancer Inst.*, **34**, 241.

Flanagan, S. P. (1966) " Nude ", a New Hairless Gene with Pleiotropic Effects in the Mouse. *Genet. Res.*, **8**, 295.

Gazzolo, L., de-Thé, G., Vuillaume, M. & Ho, H. C. (1972) Nasopharyngeal Carcinoma. II. Ultrastructure of Normal Mucosa, Tumor Biopsies, and Subsequent Epithelial Growth *in Vitro*. *J. natn. Cancer Inst.*, **48**, 73.

Giovanella, B. C. & Stehlin, J. S. (1973) Heterotransplantation of Human Malignant Tumors in " Nude " Thymus-less Mice. I. Breeding and Maintenance of " Nude " Mice. *J. natn. Cancer Inst.*, **51**, 615.

Giovanella, B. C., Stehlin, J. S. & Williams, L. J. (1974) Heterotransplantation of Human Malignant Tumors in " Nude " Thymus-less Mice. II. Malignant Tumors Induced by Injection of Cell Cultures derived from Human Solid Tumors. *J. natn. Cancer Inst.*, **52**, 921.

Hirsch, N. S., Phillips, S. M., Solnik, C., Black, P. H., Schwartz, R. S. & Carpenter, C. B. (1972) Activation of Leukemia Viruses by Graft-*versus*-Host and Mixed Lymphocyte Reactions *in Vitro*. *Proc. natn. Acad. Sci. U.S.A.*, **69**, 1069.

Kindred, B. (1971) Antibody Response in Genetically Thymusless Nude Mice Injected with Normal Thymus Cells. *J. Immunol.*, **107**, 1291.

KLEIN, G., GIOVANELLA, B. C., LINDAHL, T., FIALKOW, P. J., SINGH, S. & STEHLIN, J. S. (1974) Direct Evidence for the Presence of Epstein–Barr Virus DNA and Nuclear Antigen in Malignant Epithelial Cells from Patients with Poorly Differentiated Carcinoma of the Nasopharynx. *Proc. natn. Acad. Sci. U.S.A.*, **71**, 4737.

PANTELOURIS, E. M. (1968) Absence of Thymus in a Mouse Mutant. *Nature, Lond.*, **217**, 370.

POVLSEN, C. O., FIALKOW, P. J., KLEIN, E., KLEIN, G., RYGAARD, J. & WIENER, F. (1973) Growth and Antigenic Properties of a Biopsy-derived Burkitt's Lymphoma in Thymus-less (*Nude*) Mice. *Int. J. Cancer*, **11**, 30.

PRICE, P. J., ARNSTEIN, P., SUK, W. A., VERNON, M. L. & HUEBNER, R. J. (1975) Type-C RNA Viruses of the NIH Nude Mouse. *J. natn. Cancer Inst.*, **55**, 1231.

REED, N. D. & JULIA, J. W. (1972) Immune Response of Congenitally Thymusless Mice to Heterologous Erythrocytes. *Proc. Soc. exp. Biol. Med.*, **139**, 1234.

SHERR, C. J., LIEBER, M. M. & TODARO, G. J. (1974) Mixed Splenocyte Cultures and Graft *versus* Host Reactions Selectively Induce an " S-tropic " Murine Type C Virus. *Cell*, **1**, 55.

TODARO, G. J., ARNSTEIN, P., PARKS, W. P., LENNETTE, E. H. & HUEBNER, R. J. (1973) A type-C Virus in Human Rhabdomyosarcoma Cells after Inoculation into NIH Swiss Mice Treated with Antithymocyte Serum. *Proc. natn. Acad. Sci. U.S.A.*, **70**, 859.

TRALKA, T. S., RABSON, A. S. & HANSEN, C. T. (1975) C-type Virus Particles in a Cell Line from a Lymphosarcoma of a Nude Mouse. *J. natn. Cancer Inst.*, **55**, 197.